# Comparative Analysis of Complete Mitochondrial Genomes of Three *Gerres* Fishes (Perciformes: Gerreidae) and Primary Exploration of Their Evolution History

**DOI:** 10.3390/ijms21051874

**Published:** 2020-03-09

**Authors:** Huiting Ruan, Min Li, Zhenhai Li, Jiajie Huang, Weiyuan Chen, Jijia Sun, Li Liu, Keshu Zou

**Affiliations:** 1 Joint Laboratory of Guangdong Province and Hong Kong Region on Marine Bioresource Conservation and Exploitation, College of Marine Science, South China Agriculture University, Guangzhou 510642, China; rht_sagittarius@163.com (H.R.);; 2 Key Laboratory of Open-Sea Fishery Development, Ministry of Agriculture and Rural Affairs, South China Sea Fisheries Research Institute, Chinese Academy of Fishery Sciences, Guangzhou 510300, China; limin@scsfri.ac.cn; 3 Guangdong Laboratory for Lingnan Modern Agriculture, South China Agriculture University, Guangzhou 510642, China

**Keywords:** *Gerres filamentosus*, *Gerres erythrourus*, *Gerres decacanthus*, mitochondrial genome, phylogeny, divergence time

## Abstract

Mitochondrial genome is a powerful molecule marker to explore phylogenetic relationships and reveal molecular evolution in ichthyological studies. *Gerres* species play significant roles in marine fishery, but its evolution has received little attention. To date, only two *Gerres* mitochondrial genomes were reported. In the present study, three mitogenomes of *Gerres* (*Gerres filamentosus*, *Gerres erythrourus*, and *Gerres decacanthus*) were systemically investigated. The lengths of the mitogenome sequences were 16,673, 16,728, and 16,871 bp for *G. filamentosus*, *G. erythrourus*, *and G. decacanthus*, respectively. Most protein-coding genes (PCGs) were initiated with the typical ATG codon and terminated with the TAA codon, and the incomplete termination codon T/TA could be detected in the three species. The majority of AT-skew and GC-skew values of the 13 PCGs among the three species were negative, and the amplitude of the GC-skew was larger than the AT-skew. The genetic distance and Ka/Ks ratio analyses indicated 13 PCGs were suffering purifying selection and the selection pressures were different from certain deep-sea fishes, were which most likely due to the difference in their living environment. The phylogenetic tree was constructed by molecular method (Bayesian Inference (BI) and maximum Likelihood (ML)), providing further supplement to the scientific classification of fish. Three *Gerres* species were differentiated in late Cretaceous and early Paleogene, and their evolution might link with the geological events that could change their survival environment.

## 1. Introduction

Mitochondrial genomes (mitogenome) have become a powerful molecule marker to explore phylogenetic analysis and taxonomic diagnosis in ichthyological studies because of its simple genetic structure, maternal inheritance, fast evolutionary rate, high specificity and easy detection [1]. The mitochondrial species-specific DNA fragments, such as ribosomal RNA (12S and 16S), cytochrome b (Cytb) and cytochrome c oxidase I (COI) are usually used for fish species identification [2,3]. However, individual mitochondrial gene fragments have showed a poor performance in exploring the phylogenetic relationship among divergent species and distinguishing certain congeneric species [4,5]. Even though the COI which is viewed as a ‘DNA barcode’ cannot identify some closely related fishes due to the slow evolution and insufficient nucleotide mutation rate [6,7]. Furthermore, either the non-coding region (control region) or Cytb shows weak abilities to solve relationship in a rapid radiation [8]. Consequently, the complete mitochondrial genome with greater sequences data can provide more insights and better resolution than single mitochondrial sequences in taxonomic level [4].

Mitogenome is typically circular in vertebrates, generally composed of protein-coding genes (PCGs), transfer RNA (tRNA) genes, ribosomal RNA (rRNA) genes, and one non-coding control region (D-loop) with a whole length of approximately 15–20 kb [9]. As the most abundant group of vertebrates, fish has a mitochondrial genome that is similar in composition and structure to most vertebrates [10]. Generally, the arrangement of mitochondrial genes is extremely compacted and highly conserved, but the information offered by mitogenome is distinctive among different species [11]. However, most researches on fish mitochondrial genome have just simply described the gene structure of single species without thorough comparisons so that the understanding of fish mitochondrial genomes was not deep enough [12].

Gerreidae fishes belong to Perciformes, commonly known as mojarras or silver biddies, and comprises eight genera: *Gerres* (with 28 species), *Eucinostomus* (10 species), *Gerres* (six species), *Diapterus* (four species), *Parequula* (two species), *Pentapion* (monotypic), *Deckertichthys* (monotypic), and *Ulaema* (monotypic) [13]. As marine carnivorous species, Gerreidae fishes mainly inhabit tropical and subtropical coastal waters that are frequently affected by freshwater [14,15]. Gerreidae fishes not only play important roles in the domain of marine commercial fishes, but also have become one of the most representative groups in the tropical and subtropical aquatic ecosystems [16]. The data displayed by Fisheries and Aquaculture Department of Food and Agriculture Organization (FAO) have showed that annual capture production of the Gerreidae family has increased from 242 t in 1950 to 12,674 t in 2017, and the genus of *Gerres* is the domination of the fishery capture with higher exploitation [17]. 

Recently, related studies on genus *Gerres* mainly concentrate on morphology and description of new species [18,19,20,21], and the taxonomy of Gerreidae still remain controversial because of its taxonomic disordered history [22,23]. With critically economic and ecological status in the marine fishery, molecular biology, and evolution on *Gerres* have received little attention. To date, 178 complete mitochondrial genomes of Perciformes have deposited in the MitoFish GenBank, but only two mitogenomes for *Gerres* fishes (*G. oyena* and *G. filamentosus*) were sequenced [24]. 

In the present study, the complete mitogenomes of three *Gerres* species (*G. filamentosus*, *G. erythrourus*, and *G. decacanthus*) which were widely distributed in the South China sea [25], were sequenced. The characteristics were described and compared with each other to evaluate the variation and conservation in mitochondrial genome among *Gerres* species. The relative synonymous codon usage (RSCU) and AT skew value of PCGs were analyzed to better understanding the functional inference of related genes. Moreover, phylogenetic analysis among *Gerres* species and related species in Perciformes were conducted to investigate the kinship between them. The evolution rate and divergence time of the three species were also calculated to evaluate the adaptive capacity to environment, the situation of selection pressures, and how the environment influences their differentiation. 

## 2. Results and Discussion

### 2.1. Genome Structure and Nucleotide Composition

**Figure 1:**
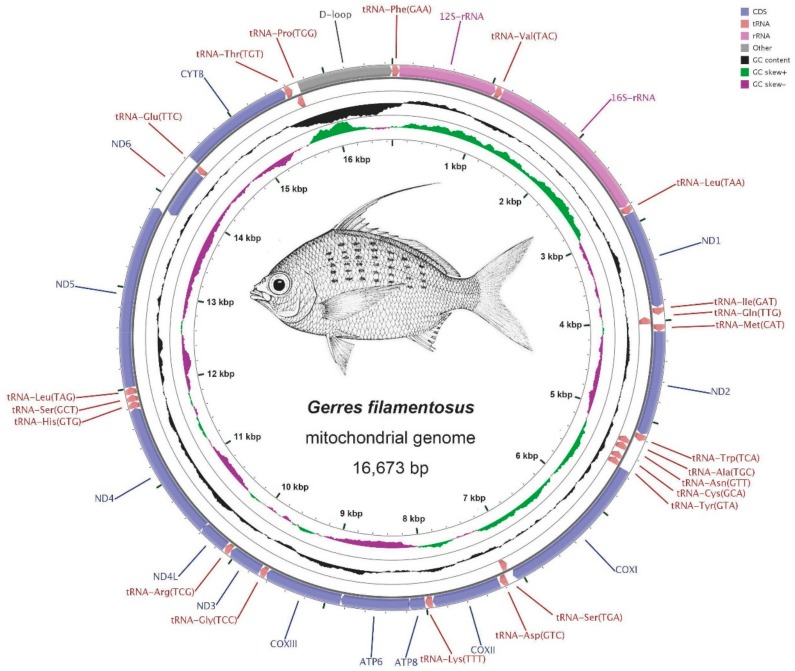

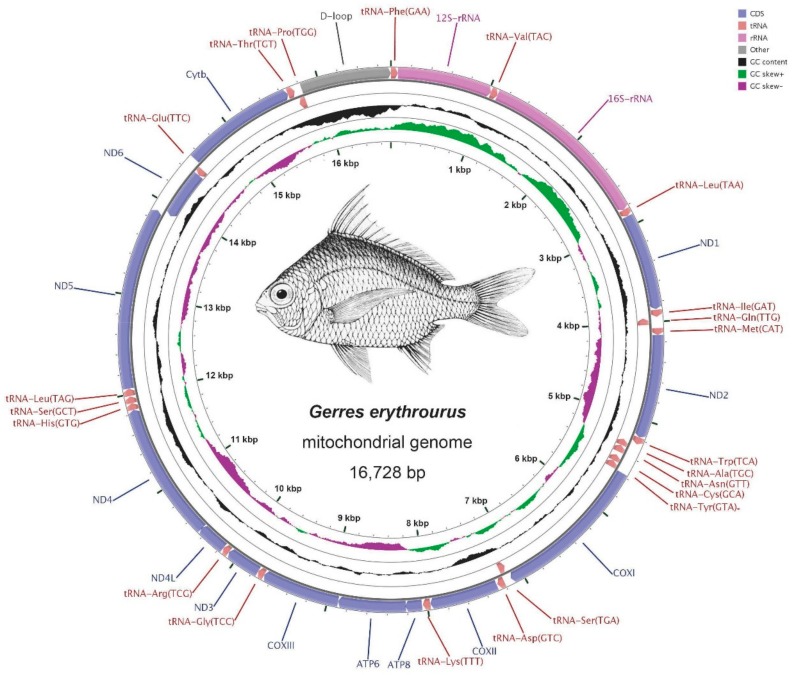

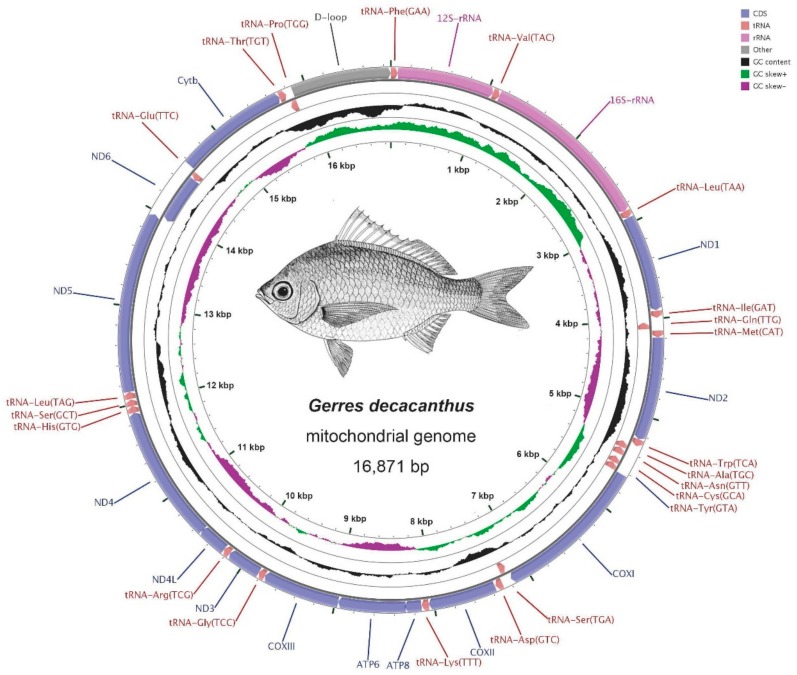
Circular map of the mitogenome of *G. decacanthus*, *G. erythrourus*, and *G. filamentosus*. Genes encoded on the heavy or light strands are shown outside or inside the circular gene map, respectively.

The mitogenome sequences of *G. filamentosus*, *G. erythrourus*, and *G. decacanthus* were 16,673, 16,728, and 16,871 bp in length, respectively. The mitogenome contained 13 PCGs (ATP6, ATP8, Cytb, COXI-III, ND1-6, and ND4L), two rRNA genes (12SrRNA and 16SrRNA), 22 tRNA genes and one D-loop region (Table 1 and Figure 1). Among these genes, ND6 and eight tRNA genes (Gln, Ala, Asn, Cys, Tyr, Ser, Glu, and Pro) were encoded on the light strand (L-strand), and the others were located on the heavy strand (H-strand). The D-loop was located between tRNA-Pro and tRNA-Phe gene as well as other vertebrates (Figure 1) [11]. The gene structure and arrangement of these species were identical with other vertebrates mitogenomes, without gene rearrangement, which were always detected in fungus and insects which may be relate to the characteristics of species’ life history [26,27].

The GC content of each gene varied from 30.43% to 60.56%, and the highest was found in tRNA-Trp of *G. decacanthus*. In addition, the richest AT content regions were detected in the D-loop of *G. filamentosus*, tRNA-Arg of *G. erythrourus*, and tRNA-His of *G. decacanthus*, respectively (Table 1). The entire base composition of the three mitogenomes in H-strand was similar with AT content varied from 52.83% to 53.71% (Table 2). The G content of the three species was low with an obvious bias against G. Besides, the content of A and C exhibited high values at the third codon position, indicating that the codon usage preferred A and C at this position (Table 2). The content of T was the highest in the second codon position, which might be the reason of hydrophobic nature of the proteins [28].

**Figure 2:**
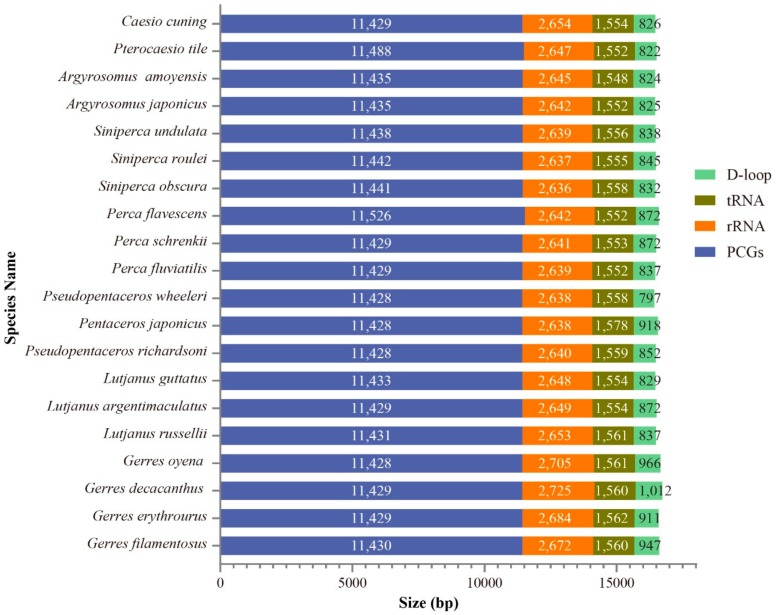
The length of protein-coding genes (PCGs), tRNAs, rRNAs, and control regions among 21 Perciformes mitogenomes.

The lengths of PCGs, tRNAs, rRNAs, and control regions for the three *Gerres* species and other Perciformes species were compared in Figure 2. The maximum length diversification was detected in D-loop, and its variation was regarded as the responsible for the differences of whole mitogenomes length [12]. Besides, the D-loop length in *Gerres* was longer than in other species, and the length of *G. decacanthus* was the highest resulted in its longest mitogenome. However, the primary sequences of D-loop seem to play minor roles in regulatory function, as the region reveals wide variability across species even their relationship were close [29]. There was a large length variation between *G*. *oyena*, *G. filamentosus*, *G. erythrourus*, and *G. decacanthus*, which belong to the same genus. The rapid variation of D-loop seems to provide some information for species revolution, but its internal mechanism needs more data and deeper examinations.

### 2.2. RNA Genes

There were 22 tRNA genes observed in mitogenomes of *G. filamentosus*, *G. erythrourus*, and *G. decacanthus* with 67–72 bp in lengths (Table 1). Both Leu (TAA, TAG) and Ser (GCT, TGA) were determined by two types of anticodons, while others were determined by only one type. Besides, 21 tRNAs displayed a canonical cloverleaf secondary structure, while tRNA-Ser (GCT) formed a simple loop missing dihydrouridine arm (D-arm). The structure of absence D-arm in tRNA-Ser has been treated as a common trait of fish mitogenomes, and it invariably transformed the recognition potential of tRNA-Ser [30]. Furthermore, the stem region of tRNA in the study contained lots of noncomplementary and T–G base pairs. In mitochondrial tRNA genes, stem mismatches seem to be a universal phenomenon and could be repaired through post-transcriptional editing [31]. The most reasonable explanation was that mitogenome was unaffected by the recombination process, and therefore allowed existence of base mismatches which might be helpful for eliminate deleterious mutations [32].

The small and large rRNA genes were recognized in the H-strand, ranging from 752 to 780 bp and 1960 to 1978 bp in length, respectively (Table 1). The AT content of the rRNA genes were 53.22% in *G. filamentosus*, 52.79% in *G. erythrourus*, and 52.95% in *G. decacanthus*, respectively, which was slightly lower than other bony fishes [33]. Defining the boundaries of rRNA genes seems to be more difficult than the PCGs which had functional annotation features [34]. Therefore, the boundaries of the genes could be inferred by assuming that there was no overlap or gap between contiguous genes.

### 2.3. Intergenic Spacers and Overlapping

Spacers in animals’ mitochondrial genes were short, and can be used for evolutionary studies due to quickly changing ratio than gene regions [35]. There were 13, 15, and 12 small intergenic spacers (IGS) region in *G. filamentosus*, *G. erythrourus*, and *G. decacanthus* totaling 87, 157, and 167 bp respectively identified (Table 1). The length of each IGS sequences ranged from 1 to 40 bp, with the longest located between tRNA-Ile and tRNA-Gln on the L-strand in *G. decacanthus*. The number and the size of IGSs was one of the reasons for mitogenome length variation [26]. The size of IGS in *G. erythrourus* and *G. decacanthus* were larger than *G. filamentosus*, wherefore the complete mitogenomes length of the former were longer than the later. Besides, the IGS situated between tRNA-Asn and tRNA-Cys representing initiation signals for replication of L-strand (O_L_) in the length of 35 bp to 37 bp. The O_L_ was always found in the intergenic region between two conserved genes [36], and the three mitogenomes were discovered. The O_L_ was usually observed between tRNA-Asn and tRNA-Cys in bony fishes, and the secondary structure that is folded into a stable stem-loop was the main feature of O_L_ [37,38].

The mitogenomes also contained overlapping regions. Five, three, and four overlap sites totaling 23, 15, and 22 bp were found in *G. filamentosus*, *G. erythrourus*, and *G. decacanthus*, respectively (Table 1). In bony fishes, the overlapping of PCGs often meant that transcripts were partially shared between abutting regions, and most reading-frames overlaps were discovered in ATP8-ATP6, ATP6-COXIII, ND4L-ND4, and ND5-ND6. The gene overlapping discovered in the same region might suggest recent common ancestry and a putative genera-specific pattern [39]. Gene overlap was one reason for mitochondrial genome compact, and the smaller mitochondrial genomes pass to offspring more frequently than the larger ones [40]. However, selection was responsible for genomes size variation, relating to adapt new environment [38,40]. The same overlapping regions were detected in the *Gerres* species, indicating they might have their own mechanism to cope with the environment.

### 2.4. Protein Coding Genes (PCGs)

The 13 PCGs of *G. filamentosus*, *G. erythrourus*, and *G. decacanthus* were 11,429 to 11,430 bp in length, accounting for 67.74% to 68.56% of the whole mitochondrial genome (Table 2). The 12 PCGs were encoded on the H-strand, only the ND6 were expressed on the L-strand in the mitogenomes. Three initiation codons (ATG, ATA, and GTG) were detected, and the ATG was the most common initiation codon in the mitochondrial genomes of three species. Except for the COXI, ATP6, and ND4 genes, all PCGs used ATG as initiation codon in the study (Table 1). Eight complete termination codons for *G. filamentosus* and six for *G. erythrourus* and *G. decacanthus* were detected (Table 1). And the incomplete termination codons (TA or T) were discovered. TA was detected in ND2, ATP6, COXIII, and ND5, while T was detected in COXII, ND3, ND4, and Cytb. These genes were followed by a gene encoded on the same strand that allowed transcription to terminate without complete codons [41]. The existence of incomplete termination codons was common in fish mitogenomes and could be accomplished by the addition of a poly A tail during RNA processing [42].

**Figure 3:**
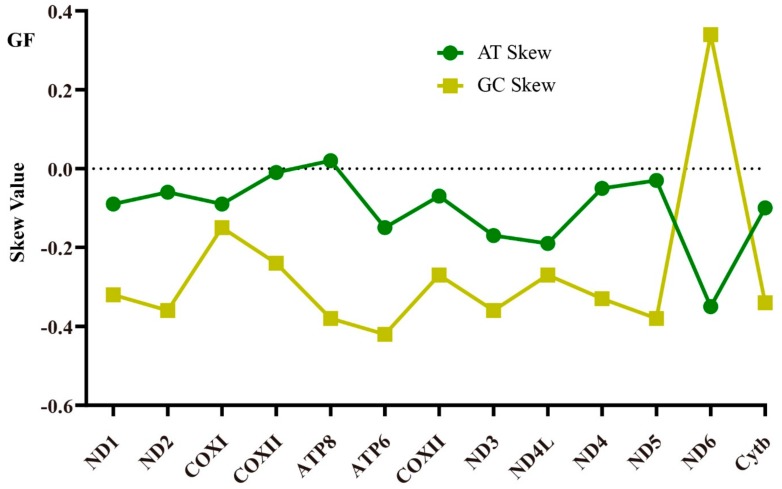

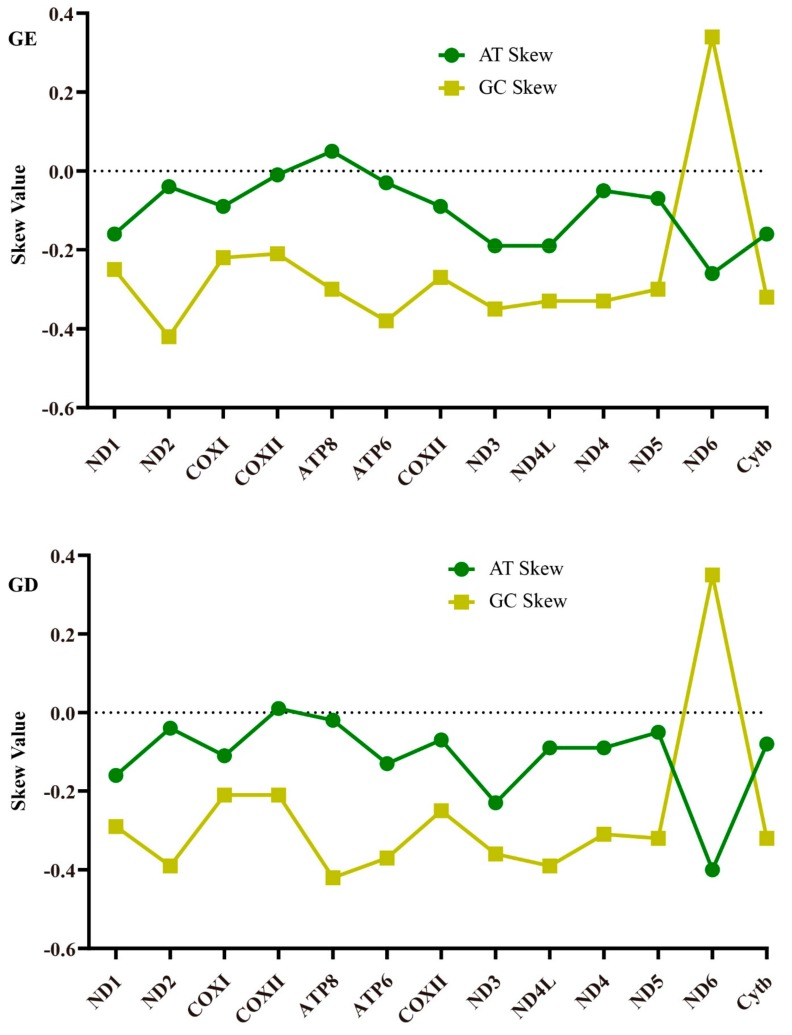
The AT/GC skew value of 13 PCGs of for *G. filamentosus* (GF), *G. erythrourus* (GE), and *G. decacanthus* (GD).

In DNA sequences, AT-skew and GC-skew was considered as a potential indicator to measure strand asymmetry and the patterns of nucleotide composition [43]. The majority of the AT-skew values and GC-skew values of the 13 PCGs among the three species were negative, demonstrating base T and C were more plentiful than A and G (Figure 3). In many cases, the amplitude of the GC-skew is larger than the AT-skew, and it is not statistically significant [11,44]. Here, the absolute value of GC-skew was indeed larger than AT-skew, which conformed to conventional preferences that GC-skew was more obvious. The lowest AT-skew and highest GC-skew value were all found in ND6, and it was the only gene displayed positive value in the GC-skew curve, which was consistent with other studies [33,43,44]. Nucleotide skew might be attributed to the equilibrium between mutation pressure and selection pressure during replication and transcription, providing a potential direction for gene replication [27,45]. ND6 had larger fluctuation in AT/GC-skew value, suggesting that the selection and mutational pressure on it might be significantly different from other genes.

### 2.5. Usage of Mitogenome Codon

**Figure 4:**
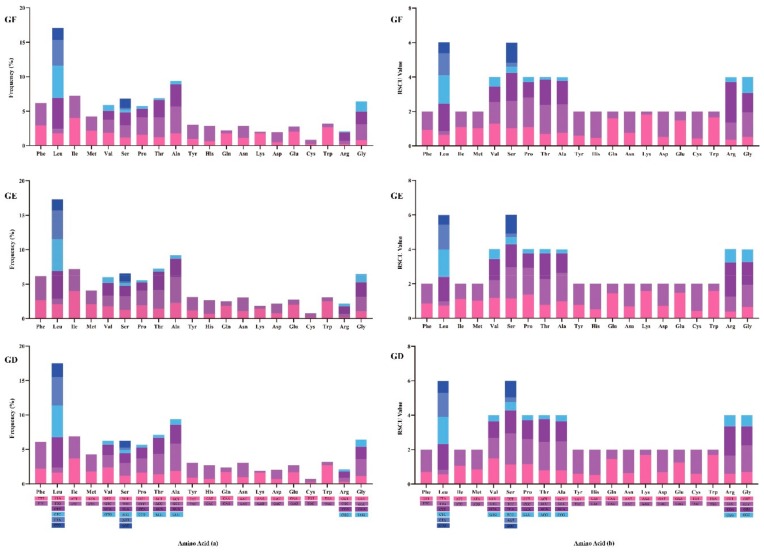
Codon frequency (**a**) and Relative Synonymous Codon Usage (**b**) of mitochondrial genome for *G. filamentosus* (GF), *G. erythrourus* (GE), and *G. decacanthus* (GD).

The amino acid, Leu, had the highest value of codon usage, which was utilized by six different codons. Cys was the least used amino acids and were encoded by only two codons (Figure 4a). The using frequency of each amino acid in *G. filamentosus*, *G. erythrourus,* and *G. decacanthus* was relatively identical.

RSCU was also used to assess mitochondrial gene codon usage. When the RSCU value = 1, it indicated that the frequency of use of codons had no different with other degenerate codons; when the RSCU value >1, it represented the codon was used more frequently [46]. The RSCU value of all amino acids in the three species were not equal to 1, implying that the usage of each amino acid had varying degrees of bias (Figure 4b). The biases of codon usage were significant in the mitochondrial genomes of different species, and it made the gene under different selection pressure and could predict the gene function [47,48]. The identical RSCU values for each amino acid in three species suggested the gene function in family Gerreidae might similar. They displayed more quantity of NNA and NNC, echoing with the result of nucleotide composition analysis of third position that was preference A and C (Table 2). Mutation pressure, genetic drift and natural selection were the main elements affecting codon bias [49]. In addition, GC contents at the third codon position, gene expression levels and gene length also were related to the codon bias [47,48]. The main evolutionary force led to high content of A + T or G + C was the mutation pressure in animals. Comparing with the low GC content gene, higher GC content of third codon position seemed easier methylated and caused mutations [50].

### 2.6. Variations, Genetic Distance, and Evolution Rates of PCGs

**Figure 5:**
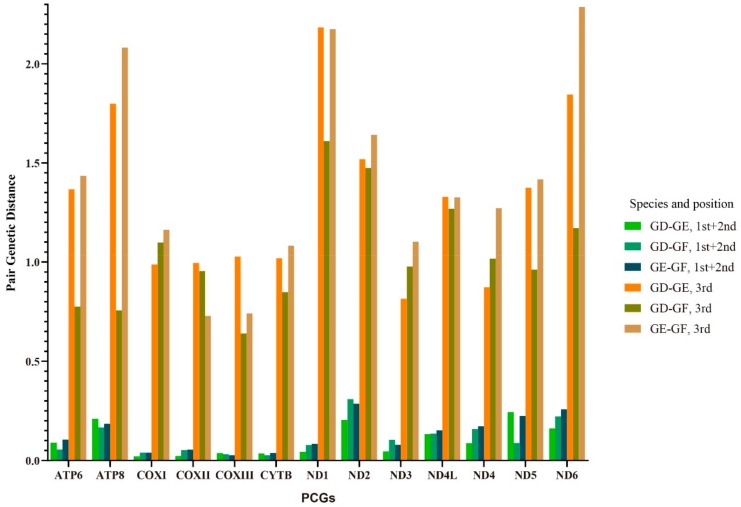
The pair genetic distances of 13 PCGs between *G. filamentosus*, *G. erythrourus,* and *G. decacanthus*. The values were calculated based on the first and second nucleotide position, and on the third nucleotide position, respectively.

The genetic distance could be used to evaluate different mutation pressures among genes [51]. The pairwise genetic distances (p-distance) were calculated to reveal the sequence conservation and divergence of the PCGs among the *Gerres* species (Figure 5). The genetic distance at the third nucleotide position was obviously higher than the first and second nucleotide position, indicating that the evolution of the third position was faster than the first and the second. The highest p-distance were found in ND1 (2.184, 1.610) and ND6 (2.286) at the third nucleotide of codons, while explored in ND2 (0.309, 0.286) and ND5 (0.243) base on the first and second nucleotide position (Figure 5). The COXI-III and Cytb genes had low genetic distance in both first + second and third analysis. ND1, ND2, and ND6 genes might have high evolutionary rates among the three species, while COXI-III and Cytb were low.

The value of nonsynonymous substitution (Ka)/synonymous substitution (Ks) is a common indicator to assess selective pressure and evolutionary relationships of species in molecular studies [52]. Ka/Ks < 1, Ka/Ks = 1, and Ka/Ks > 1 were represented purifying selection, neutral mutation and positive selection, respectively [53]. All 13 PCG genes were under strong purifying selection with Ka/Ks values below 1 (Figure 5). The result was different from deep-sea fishes, where most genes exhibited positive selection or convergent/parallel signals with the exception of ND4L and ND5 [54]. One of the reasons might be the different living environment between them. The basic characteristics of genome evolution depended on random genetic drift and mutation pressure that closely connected with the environment [55]. The deep-sea fishes inhabited in the condition of oxygen deficiency, food lacked, no sunlight and extreme cold, while the *Gerres* species survived in the warm coastal waters [15,16,54]. Positive selection usually related to the adaptation of new environments and the development of new function, and most nonsynonymous mutations were disadvantage [56,57]. The Ka/Ks values in *Gerres* species showed they were under purifying selection, indicating that the environment variation was not great enough to change their genetic function.

**Figure 6:**
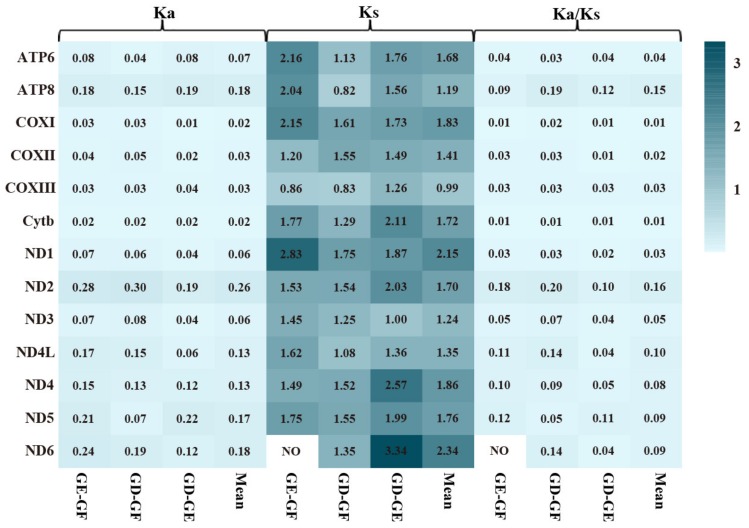
The rates of non-synonymous substitutions and synonymous substitutions for each PCG in pairwise mitochondrial genome of *G. filamentosus* (GF), *G. erythrourus* (GE), and *G. decacanthus* (GD).

ATP8 and ND2 genes had high Ka/Ks (mean: 0.15, 0.16) values across three *Gerres* mitogenomes comparing to other genes, while COXI and Cytb genes were low (Figure 6). Low mutation rates tended to occur on highly expressed genes due to DNA repair mechanisms [58]. Compare to other genes, the COXI and Cytb showed low Ka/Ks representing a low mutation rate, indicating that they may have higher expression level.

**Figure 7:**
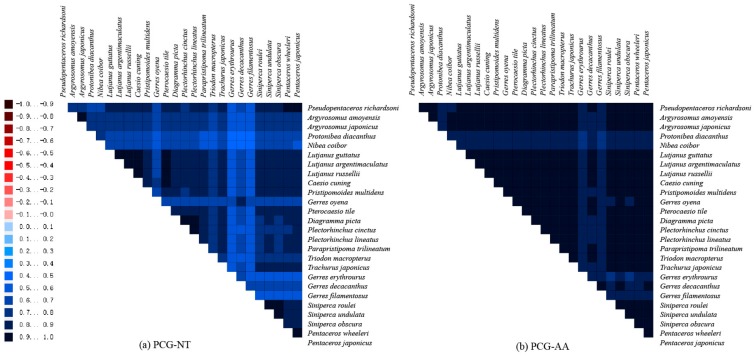
(**a**) AliGROOVE analysis of 13 PCGs for 26 mitochondrial genomes considering their nucleotide composition. (**b**) AliGROOVE analysis based on amino acid composition of 13 PCGs. The obtained mean similarity score between sequences is represented by a colored square. The site scores are ranging from −1, indicating great difference in sequence composition (red coloring), to +1, indicating similarity to other sequence composition (blue coloring).

The AliGROOVE analysis of 13 PCGs showed that there were no strongly divergent patterns among 26 species and all sites displayed positive scores. Besides, the Gerreidae exhibited higher heterogeneity than other families (Figure 7). The site score of nucleotides dataset was lower than the amino acid dataset, indicating that the degrees of heterogeneity of the PCG-NT datasets were higher than PCG-AA datasets. Generally, high divergences between different taxa suggested that species was not robustly placed or might be misplaced on phylogenetic trees [59]. From the PCG-NT or PCG-AA datasets, the heterogeneity of most pairwise comparisons was low with site score above 0.5. The low heterogeneity of pairwise comparisons demonstrated that the two datasets were applicable for further phylogenetic studies [60].

### 2.7. Phylogenetic and Divergence Times

**Figure 8:**
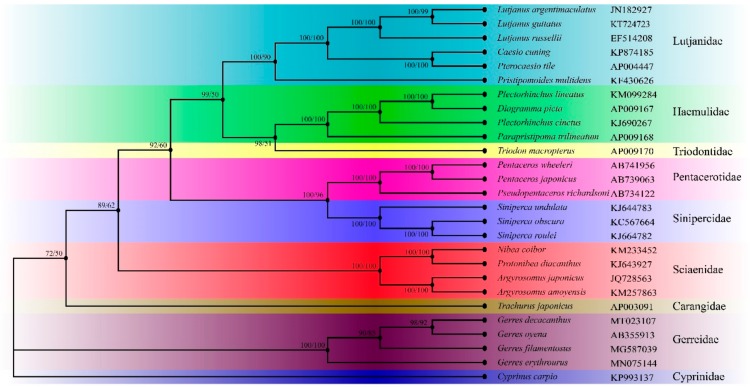
Phylogenetic tree of 27 Perciformes species constructed by Bayesian Inference (BI) and maximum Likelihood (ML) methods base on concatenated sequences of 13 PCGs. *Cyprinus carpio* was used as the outgroup. Numerals at nodes are Bayesian posterior probabilities (left) and bootstrap support values (right), respectively.

The phylogenetic analysis contained 27 species from nine families (Lutjanidae, Haemulidae, Triodontidae, Pentacerotinae, Sinpercidae, Sciaenidae, Carangidae, Gerreidae, and Cyprinidae) (Figure 8). The phylogenetic trees inferred by two methods generated identical topologies and had formidable values of intermediate bootstrap and post probability. It showed that each family gathered together and separated with other families, while Gerridae species were clustered to be one branch without sister lineage. It was consistent with the traditional morphological classification, indicating that the morphological phenotypes of fish were closely related to genetic background [61]. The phylogenetic tree also revealed that the *G. decacanthus* had the closest relationship with *G. oyena* and the farthest with *G. erythrourus* in family Gerridae.

**Figure 9:**
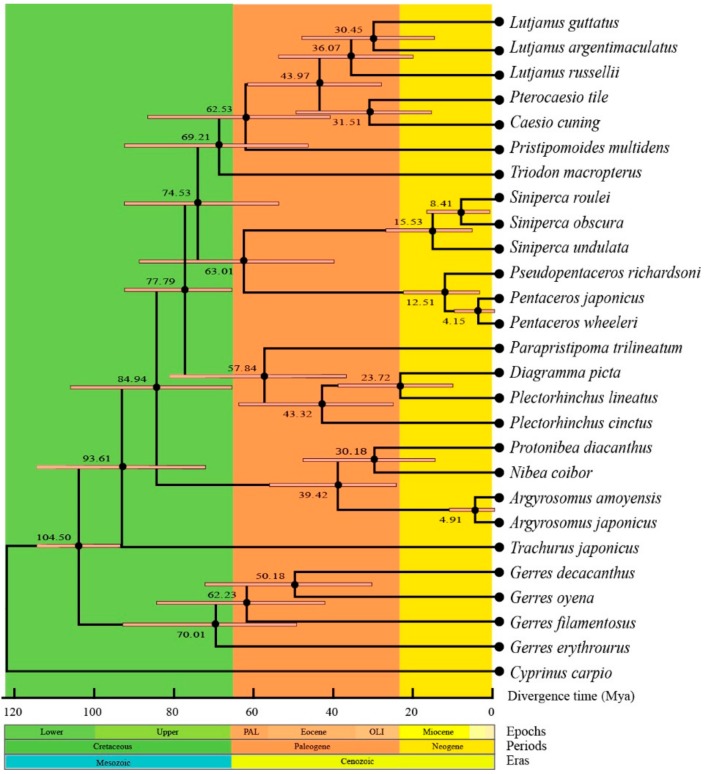
Divergence time analysis of 27 fish species base on Maximum likelihood topology using concatenated sequences of 13 PCGs. Numbers near the nodes indicated the estimated divergence time (Mya).

The present divergence time calculations showed that the initial of the *Gerres* species was separated around 104.5 million years ago (Mya) (Figure 9). In family Gerreidae, the differentiation time between *G. erythrourus* and other *Gerres* species was the earliest (70.01 Mya). However, the *G. decacanthus* and *G. oyena* had latest differentiation time (50.18 Mya). There were 7.78 million gaps between *G. erythrourus* and *G. filamentosus*, and 12.05 between *G. filamentosus* and *G. decacanthus*. The accumulation of changes in genetic composition resulted species reproductive isolation and evolution, indicating evolution was a long-term process as shown in the earlier study [57]. And the change of genetic structure might relate to mutations, recombination, selection, drift, migration, and isolation. However, the occurrence of geological events was the main reason for migration and isolation [62,63]. The divergence time of *Gerres* species could trace back to the late Cretaceous (66–145 Mya). At that time, many mountains were formed, angiosperms began to appear and shale were extensively deposit to the ocean and the Oceanic Anoxic Event 2 occurred [64]. The geological events might change the habitats of fish and their genetic structure might induce differentiation. Thus, the Gerreidae family differentiated in the period. Besides, *G. filamentosus* differentiation from other species was around 62.23 Mya, and *G. decacanthus* and *G. oyena* were differentiated around 50.18 Mya. Cenozoic-Paleogene, approximately occurred at 2.4–65 Mya, a period with significantly shrank of transgressive range in the continent and appeared of marine sediments in the marginal areas of China [65]. Geographical isolation caused by geological movements might provide sufficient environmental conditions for divergence of fish, while high aquatic biological productivity caused by marine sedimentation could offer food sources for growth. In the present study, the divergence of most species concentrated on the Cenozoic-Paleogene, and should be closely related to these geological events.

## 3. Materials and Methods

### 3.1. Samples and DNA Extraction

Wild specimens of *G. filamentosus* (June, 2017, E113°36′, N22°74′), *G. erythrourus* (March, 2018, E113°30′, N22°25′), and *G. decacanthus* (March, 2018, E113°30′, N22°25′) were collected in Pearl River Estuary, and were deposited in the South China Agriculture University, Guangzhou, China. One individual of each species was used for DNA extraction. The dorsal muscle was collected and the genomic DNA was extracted by the TIANamp Marine Animals DNA Kit (TIANGEN, Beijing, China) following the manufacturer’s protocol, except that the final step was eluted with sterilized water instead of TE. The integrity of DNA samples was firstly characterized by 0.8% gel electrophoresis and UV spectroscopy. DNA concentration and purity were measured by NanoDrop 2000 spectrophotometer (Thermo Scientific, Wilmington, USA) and Qubit 2.0 Flurometer (Invitrogen, California, USA). Qualified DNA samples were stored at −20 °C for the next experiment. All animal experiments were conducted in accordance with the guidelines and approval of the Animal Research and Ethics Committees of South China Agricultural University.

### 3.2. Library Construction and High-Throughput Sequencing

High-quality DNA samples were randomly broken into fragments with the length of 350 bp for paired-end sequencing, and the DNA libraries were constructed according to the standard procedure of Illumina DNA library construction. The quality of the library was control by qPCR method and Agilent 2100 Bioanalyzer ( Agilent, California, USA). The quality-qualified DNA library was run on an Illumina HiSeq 4000 instrument (Illumina, California, USA) with paired-end reads of 150 bp, and the sequencing data of per sample was not less than 2 GB.

### 3.3. Sequence Assembly, Annotation, and Analysis

The original sequences obtained by Illumina HiSeq 4000 sequencing were filtered to get high-quality sequences, with the following principle: when the content of N in any sequencing reads exceeded 10% of the number of read bases or any sequencing reads containing low-quality (Q ≤ 5) bases exceeded 50%, the paired reads were removed. The obtained high-quality fragments were aligned with Gerridae mitochondrial genomes on NCBI to remove sequence repeats and inaccurate sequencing, and then assembled by SPAdes v.3.5.0 [66] software to obtain the complete circular mitochondrial genome. The preliminary annotation of mitochondrial genome were used MitoFish [24] (http://mitofish.aori.u-tokyo.ac.jp/) and ORF Finder (https://www.ncbi.nlm.nih.gov/orffinder/). The protein-coding regions and ribosomal genes were determined by align with the reported mitochondrial genomes of close related species base on the methods of Blastl and Blastn (https://blast.ncbi.nlm.nih.gov/Blast.cgi). The complete mitogenomes of *G. filamentosus*, *G. erythrourus* and *G. decacanthus* were uploaded to GenBank with accession number MG587039, MN075144, and MT023107, respectively.

Mitochondrial gene structure maps were drawn using CGView Server [67] (http://stothard.afns.ualberta.ca/cgview_server/). The secondary structure of tRNAs were obtained by ARWEN (Version1.2) [68], and verified again by tRNAscan-SE 2.0 [69] (http://lowelab.ucsc.edu/tRNAscan-SE/) if any tRNA structure was abnormal. Sequence length, nucleotide composition and codon usage were calculated via DNAStar. The following formulas were used to calculate the values of AT/GC-skew to assess the nucleotide bias: AT-skew = (A – T)/(A + T) and GC-skew = (G – C)/(G + C). The data of RSCU were received by MEGA 7 software [70]. Based on the RSCU values, a histogram of the distribution and visualization of codon usage were drawn by software of GraphPad Prism 8.1. The AliGROOVE [71] was used to assess the heterogeneities of sequence divergence, separately for different datasets.

### 3.4. Phylogenetic Analyses

To establish evolutionary relationships among *G. filamentosus*, *G. erythrourus* and *G. decacanthus* and the related species, the complete mitogenomes of other 24 Perciformes species were downloaded from GenBank. The phylogenetic tree was constructed using concatenated sequences of 13 PCGs. The MUSCLE v.3.8.31 software [72] was utilized to perform the alignment of individual genes between multiple species and excluded the start and stop codon. The *Cyprinus carpio* (GenBank accession number: KP993137) was used as the out-group to determine the root of phylogenetic tree [44]. The Bayesian Inference (BI) and maximum Likelihood (ML) methods were applied and the optimal model for nucleotide sequences was estimated by jModelTest2.1.7 [73]. Mtmam + I + G + F captured the minimum values of Akaike Information Criteria (AIC) and was considered to be the best model for phylogenetic tree construction. The ML tree was constructed by RAxML8.1.5 software [74] with 1000 replicates of bootstrap and the BI analysis was inferred by the software of MrBayes 3.2.6 based on 10,000,000 generations [75]. The divergence time was predicted by MEGA 7.0 with the RelTime-ML method and GTR + I + G modeling [70]. The calibration of divergence times were obtained from online Time Tree database (http://www.timetree.org/) [76].

## 4. Conclusions

In the present study, mitogenome sequnences of *G. filamentosus*, *G. erythrourus*, and *G. decacanthus* were obtained by high-throughput sequencing. Their mitogenomes were with a total length of 16,673 bp in *G. filamentosus*, 16,728 bp in *G. erythrourus*, and 16,871 bp in *G. decacanthus*, respectively. Each of the mitogenome composed of 13 PCGs, 2 rRNAs, 22 tRNAs and one D-loop. Most PCGs were initiated with the typical ATG codon and terminated with TAA codon. The ratio of Ka and Ks indicated that three species were suffering a purifying selection, while the COI and Cytb showed the highest Ka/Ks values. The three *Gerres* species were differentiated in late Cretaceous and early Paleogene, and their evolution might link with the geological events that could change their survive environment. The phylogenetic tree provided further supplement to the scientific classification of *Gerres* fishes. This study could provide basis information for genetic characters, phylogenetic position and evolution profile for these fishes, which could benefit for resource management or selective breeding in fishery and aquaculture.

## Figures and Tables

**Table 1:** Summary of gene/element feature of *G. filamentosus* (GF), *G. erythrourus* (GE), and *G. decacanthus* (GD).

Gene/Element	Strand	Position Start/End			Size (bp)			GC Percentage			Codon Start/Stop			Intergenic Nucleotide* (bp)			Anti-Codon/One Letter Code
		GF	GE	GD	GF	GE	GD	GF	GE	GD	GF	GE	GD	GF	GE	GD	
tRNA-Phe	H	1/71	1/70	1/71	71	70	71	42.25%	48.57%	42.25%				0	0	0	GAA/F
12S-rRNA	H	72/1053	71/1022	72/1053	982	952	982	48.98%	48.63%	49.90%				0	0	0	
tRNA-Val	H	1054/1122	1023/1093	1054/1124	69	71	71	43.48%	43.66%	40.85%				1	0	0	TAC/V
16S-rRNA	H	1124/2813	1094/2825	1125/2867	1690	1732	1743	45.50%	46.42%	45.44%				0	0	0	
tRNA-Leu	H	2814/2887	2826/2899	2868/2941	74	74	74	52.70%	45.95%	51.35%				0	0	0	TAA/L
ND1	H	2888/3862	2900/3874	2942/3916	975	975	975	47.59%	49.44%	50.67%	ATG/TAA	ATG/TAA	ATG/TAG	7	5	6	
tRNA-Ile	H	3870/3940	3880/3951	3923/3993	71	72	71	53.52%	54.17%	60.56%				11	5	40	GAT/I
tRNA-Gln	L	3952/4026	3957/4027	4034/4104	75	71	71	44.00%	46.48%	46.48%				9	37	18	TTG/Q
tRNA-Met	H	4036/4104	4065/4133	4123/4193	69	69	71	40.58%	40.58%	43.66%				0	0	0	CAT/M
ND2	H	4105/5151	4134/5179	4194/5239	1047	1046	1047	48.42%	48.09%	52.58%	ATG/TAG	ATG/TA	ATG/TAA	0	0	0	
tRNA-Trp	H	5152/5222	5180/5251	5241/5312	71	72	72	49.30%	50.00%	54.17%				1	1	1	TCA/W
tRNA-Ala	L	5224/5292	5253/5321	5314/5382	69	69	69	40.58%	37.68%	40.58%				1	2	2	TGC/A
tRNA-Asn	L	5294/5366	5324/5396	5385/5457	73	73	73	49.32%	47.95%	49.32%				35	37	36	GTT/N
tRNA-Cys	L	5402/5468	5434/5501	5494/5560	67	68	67	49.25%	45.59%	44.78%				0	0	0	GCA/C
tRNA-Tyr	L	5469/5539	5502/5572	5561/5631	71	71	71	47.89%	46.48%	46.48%				1	1	1	GTA/Y
COXI	H	5541/7091	5574/7124	5633/7183	1551	1551	1551	46.74%	46.16%	47.32%	GTG/TAA	GTG/TAA	GTG/TAA	0	0	0	
tRNA-Ser	L	7092/7162	7125/7195	7184/7254	71	71	71	53.52%	52.11%	50.70%				3	3	29	TGA/S
tRNA-Asp	H	7166/7237	7199/7270	7284/7354	72	72	71	40.28%	45.83%	50.70%				9	10	9	GTC/D
COXII	H	7247/7937	7281/7971	7364/8054	691	691	691	46.45%	43.99%	45.30%	ATG/T	ATG/T	ATG/T	0	0	0	
tRNA-Lys	H	7938/8011	7972/8046	8055/8128	74	75	74	52.70%	50.67%	55.41%				1	8	1	TTT/K
ATP8	H	8013/8180	8055/8222	8130/8297	168	168	168	47.62%	42.26%	45.24%	ATG/TAA	ATG/TAA	ATG/TAA	−10	−7	−10	
ATP6	H	8171/8854	8216/8895	8288/8970	684	680	683	46.20%	45.29%	47.29%	ATG/TAA	ATA/TA	ATG/TA	−1	0	0	
COXIII	H	8854/9638	8896/9680	8971/9755	785	785	785	49.17%	46.50%	48.41%	ATG/TA	ATG/TA	ATG/TA	0	1	0	
tRNA-Gly	H	9639/9708	9682/9751	9756/9825	70	70	70	37.14%	42.86%	38.57%				0	0	0	TCC/G
ND3	H	9709/10057	9752/10100	9826/10174	349	349	349	50.72%	45.85%	49.00%	ATG/T	ATG/T	ATG/T	0	0	0	
tRNA-Arg	H	10058/10126	10101/10169	10175/10243	69	69	69	36.23%	30.43%	34.78%				0	0	0	TCG/R
ND4L	H	10127/10423	10170/10466	10244/10540	297	297	297	49.49%	51.52%	49.49%	ATG/TAA	ATG/TAA	ATG/TAA	−7	−7	−7	
ND4	H	10417/11797	10460/11840	10534/11914	1381	1381	1381	49.67%	49.31%	48.95%	ATG/T	GTG/T	ATG/T	0	0	0	
tRNA-His	H	11798/11866	11841/11909	11915/11983	69	69	69	36.23%	34.78%	30.43%				0	0	0	GTG/H
tRNA-Ser	H	1186711934	11910/11977	11984/12051	68	68	68	45.59%	51.47%	52.94%				4	5	5	GCT/S
tRNA-Leu	H	11939/12011	11983/12056	12057/12129	73	74	73	42.47%	45.95%	42.47%				0	1	0	TAG/L
ND5	H	12012/13850	12058/13898	12130/13968	1839	1841	1839	46.28%	46.50%	45.89%	ATG/TAA	ATG/TA	ATG/TAA	−4	10	−4	
ND6	L	13847/14368	13909/14430	13965/14486	522	522	522	46.55%	50.00%	49.23%	ATG/TAG	ATG/TAA	ATG/TAG	0	0	0	
tRNA-Glu	L	14369/14437	14431/14500	14487/14555	69	70	69	46.38%	48.57%	42.03%				4	30	19	TTC/E
CYTB	H	14442/15582	14531/15671	14575/15715	1141	1141	1141	46.45%	45.05%	46.45%	ATG/T	ATG/T	ATG/T	0	0	0	
tRNA-Thr	H	15583/15655	15672/15744	15716/15787	73	73	72	54.79%	54.79%	56.94%				−1	1	−1	TGT/T
tRNA-Pro	L	15655/15726	15746/15817	15787/15859	72	72	73	34.72%	38.89%	38.36%				0	0	0	TGG/P
D-loop	H	15727/16673	15818/16728	15860/16871	947	911	1012	34.21%	35.35%	39.03%				0	0	0	

Intergenic nucleotide*(bp): positive values indicate the interval sequence of adjacent genes, and negative values indicate the overlapping of adjacent genes. H represents heavy strand and L represents light strand.

**Table 2:** Base composition for protein-coding, tRNA, rRNA genes, and D-loop region of the mitogenomes of *G. decacanthus*, *G. erythrourus*, and *G. filamentosus*.

	*G. filamentosus*					Total Number	*G. erythrourus*					Total Number	*G. decacanthus*					Total Number
	A	T	G	C	A + T		A	T	G	C	A + T		A	T	G	C	A + T	
Complete genome	26.85	26.65	17.13	29.19	53.50	16,673	26.43	27.28	17.57	28.72	53.71	16,728	26.29	26.54	17.79	29.38	52.83	16,871
Protein-coding genes																		
first	24.76	21.24	26.41	27.59	46.00	3813	24.73	21.45	26.51	27.30	46.81	3813	24.52	20.72	26.78	27.98	45.24	3813
second	17.96	40.64	14.12	27.28	58.60	3809	18.27	13.89	40.69	27.15	58.97	3809	18.14	41.03	13.70	27.12	59.18	3809
third	29.18	23.63	10.61	35.58	52.81	3808	29.12	24.65	11.04	35.19	53.77	3805	27.48	23.86	11.93	36.73	51.34	3806
total	23.96	28.50	17.05	30.48	52.47	11430	24.04	28.93	17.15	29.88	52.97	11427	23.38	28.54	17.47	30.61	51.92	11428
tRNA	27.37	27.44	24.04	21.15	54.81	1560	29.05	25.27	21.24	24.44	54.32	1563	26.97	27.05	24.17	21.99	53.85	1560
rRNA	30.88	22.34	20.66	26.12	53.22	2672	30.66	22.13	21.80	25.41	52.79	2684	29.98	22.97	21.14	25.91	52.95	2725
D-loop	34.74	31.05	14.68	19.54	65.79	947	31.72	32.93	14.05	21.30	64.65	911	33.99	26.98	16.80	22.23	60.97	1012
